# Machine learning-based detection of label-free cancer stem-like cell fate

**DOI:** 10.1038/s41598-022-21822-z

**Published:** 2022-11-09

**Authors:** Alexis J. Chambost, Nabila Berabez, Olivier Cochet-Escartin, François Ducray, Mathieu Gabut, Caroline Isaac, Sylvie Martel, Ahmed Idbaih, David Rousseau, David Meyronet, Sylvain Monnier

**Affiliations:** 1 Cancer Initiation and Tumor Cell Identity Department, Cancer Research Centre of Lyon (CRCL) INSERM 1052, CNRS UMR5286, Centre Léon Bérard, Université Claude Bernard Lyon 1, 69008 Lyon, Villeurbanne, France; 2 Univ Lyon, CNRS, Institut Lumière Matière, Univ Claude Bernard Lyon 1, 69622 Villeurbanne, France; 3 Institut du Cerveau - Paris Brain Institute - ICM, Inserm, CNRS, AP-HP, Hôpital Universitaire La Pitié Salpêtrière, DMU Neurosciences, Sorbonne Université, Paris, France; 4 Laboratoire Angevin de Recherche en Ingénierie des Systèmes (LARIS), UMR Inrae IRHS, Université d’Angers, 49000 Angers, France; 5 Pathology Institute, Hospices Civils de Lyon, Lyon, France; 6 Neuro-oncology Department, Hospices Civils de Lyon, Lyon, France

**Keywords:** Image processing, Biomedical engineering, Computational science

## Abstract

The detection of cancer stem-like cells (CSCs) is mainly based on molecular markers or functional tests giving a posteriori results. Therefore label-free and real-time detection of single CSCs remains a difficult challenge. The recent development of microfluidics has made it possible to perform high-throughput single cell imaging under controlled conditions and geometries. Such a throughput requires adapted image analysis pipelines while providing the necessary amount of data for the development of machine-learning algorithms. In this paper, we provide a data-driven study to assess the complexity of brightfield time-lapses to monitor the fate of isolated cancer stem-like cells in non-adherent conditions. We combined for the first time individual cell fate and cell state temporality analysis in a unique algorithm. We show that with our experimental system and on two different primary cell lines our optimized deep learning based algorithm outperforms classical computer vision and shallow learning-based algorithms in terms of accuracy while being faster than cutting-edge convolutional neural network (CNNs). With this study, we show that tailoring our deep learning-based algorithm to the image analysis problem yields better results than pre-trained models. As a result, such a rapid and accurate CNN is compatible with the rise of high-throughput data generation and opens the door to on-the-fly CSC fate analysis.

## Introduction

The precise identification of cancer stem cell (CSC) phenotypes is a prerequisite to improve our understanding of their biology, patient diagnosis as well as future treatments. Until now, the specific identification of cell types was performed *a posteriori* by detecting a combination of markers^1^ and could not be easily implemented in living cells, especially in patient-derived samples. The tumorsphere formation assay is an alternative and complementary functional test based on CSC self-renewing capacity^2^. Indeed, in non-adherent culture conditions, CSCs are characterized by their ability to divide in suspension while maintaining cell-cell interactions to form structures of hundreds to thousands of cells called tumorspheres, similar to neural stem cell derived neurospheres^3^. The proportion of CSCs within a given cell population derived from either tumor samples or *in vitro* cell cultures can therefore be evaluated based on their capacity to survive as single cells and/or to reconstitute a hierarchical population of cancer cells in vitro^4^. Studying the dynamics of the earliest steps of the formation of tumorspheres at the single-cell level to determine cell fate can therefore bring new insights into the biology of CSCs while also shortening the evaluation of CSC proportions and their contribution to tumor resistance within patient-derived samples.

Labeling living biological samples is not systematically possible and, in addition, it might result in biochemical artefacts or phototoxicity, which can alter cell behavior^5^ and metabolism^6^. Brightfield microscopy is a widely available imaging technique to assess cell morphological characteristics, but its lack of specificity and contrast requires the development of dedicated image analysis tools to quantify different cellular phenotypes. Several studies have made use of image analysis algorithms to provide quantified data from brightfield images. For example, many classical computer vision algorithms (CCVA), *i.e.* based on a few sets of expert selected features, have been developed so far, such as thresholding methods for cell and vesicle segmentation^7^, or intensity projection from z-stacks for macrophages segmentation^8^. Brightfield images generally display high sample-to-sample variability and classical handcrafted methods based on few features and fixed decision rules require advanced programming skills and long development^9,10^.

Microfluidics can produce the high-throughput data needed for physiologically relevant single-cell analysis. Thanks to this approach, machine learning-based techniques can now be used for image analysis^11^. With shallow learning based algorithms (SLBA), the decision rules can be data driven instead of handcrafted by experts^12,13^. Deep learning-based algorithms (DLBAs) are revolutionary since they considerably reduce the time of algorithm development. The power of DLBAs in deciphering the complexity of image analysis problems has already been demonstrated on similar topics^14–16^. DLBAs have proven to be more efficient than CCVAs for white blood cell classification^17^, and in a classification problem of multi-cellular spheroids, while convolutional neural networks (CNNs) have outperformed SLBAs when trained on a large data set^18^. Along the same line, improving feature extraction thanks to transfer learning from pre-trained CNNs can also enhance histopathological biopsy classification^19^. Pre-trained CNNs have already provided excellent results to detect cell death or differentiation from cells grown in adherent conditions, however these cell features and geometry are very different from the problem faced in our study with cells expanding in non-adherent conditions and therefore cannot be applied straightforwardly^20–22^.

**Figure 1:**
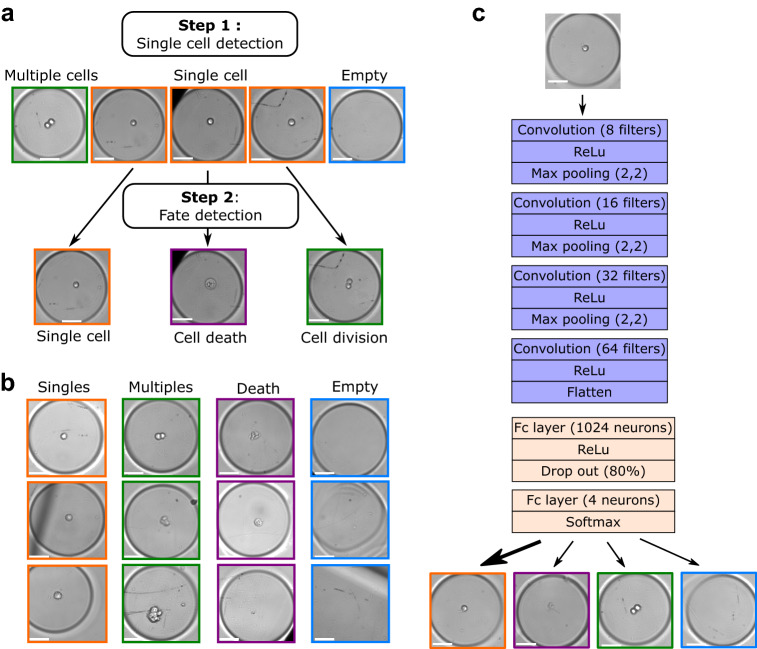
Description of our deep-learning based algorithm (DLBA). (**a**) Visual abstract of the image analysis problem. First step: detection of isolated single cells from 2D brightfield images. Second step: when a single cell was detected, the 96-h time-lapse of the corresponding well was analysed in order to detect cell division or cell death. (**b**) Examples of the different classes defined for our DLBA: single cells, multiple cells, dead cells or empty well. (**c**) Model of our optimized DLBA : 4 convolution layers before classification of images between “Singles”, “Multiples”, “Death” and “Empty”. Scale bar: 50 ${{\upmu}}$m.

Here, we provide a data driven study to automatically process brightfield images of cells growing in suspension isolated in microwells of a microfabricated chip (Fig. 1a and b)^23,24^. Despite a trivia appearance, the detection of different cell fates in these conditions is challenging as i) cells can exhibit a large variety of shapes, ii) the cell morphology is a lot more compact when grown in suspension compared to adherent conditions, iii) cells can be out-of-focus since they are cultured in non-adherent conditions, iv) images of these cells are very different from classical computer vision data base such as ImageNet^25^. To detect single cells and dynamically track their fate, we have developed a full DLBA pipeline for the high-throughput prediction of cancer cells status (division, death or quiescence) from large amount of label-free brightfield microscopy images. In this article, we describe how we tailored a DLBA to microfabricated wells to track cell-fate from two primary CSC lines. We compare our solution with a classical computer vision algorithm (CCVA), a shallow learning-based algorithm (SLBA) and several standard DLBAs. We also assayed the robustness of our DLBA on a CSC model different from the one used for the training. Taken together, we demonstrate that for this problem (defining the fate of non-adherent cells) our algorithm is more efficient than all CCVA, SLBA and pretrained large models tested as it provides equivalent or better results while allowing on-the-fly analysis of high-throughput acquisitions by saving processing time and power.

## Results

In this study, we primarily focused on addressing a problem of CSC fate prediction after being individually isolated in microwells. Our approach was divided into two phases: first, classify each microwells based on its content at the beginning of the assay: empty, alive single cell, dead single cell or more than one cell. Second, the results of the classification were linked to a 96-h time lapse analysis using a decision tree to assess the fate of the single CSCs in the microwells.

### Deep learning-based algorithm

In informational terms, we formulate the problem as an invariant to translation classification. We therefore developed a CNN adapted to our classification problem, refered to as “our DLBA” in the rest of the text. We trained our CNN on a data set which contains 17,378 annotated images of glioblastoma-derived N14-0510 CSCs imaged with a 20$\times$ magnification every 30 min, a temporal resolution selected according to the proliferation characteristics of N14-0510 cells in this culture condition. This model was based on successive layers of convolution and max pooling upstream of a first fully connected layer together with a second fully connected layer that classified images as “Singles”, “Multiples”, “Death” or “Empty” (Fig. 1c, the data sets are described in the Methods section and in the Supplemental Fig. S1a and b). Wells classified as “Multiples” can either contain two cells, sparse single cells or aggregates of cells. Classification based on brightfield images was controlled using fluorescent markers for cell numbers and cell death (Supplemental Fig. S2a and b).

We have optimized the number of layers, the number of filters per convolution layer and the kernel size: four convolution layers of 8, 16, 32 and 64 filters respectively, and a kernel size of $15 \times 15$ pixels gave the best accuracy (Supplemental Fig. S1c). Secondly, we have determined the optimal number of neurons in the first fully connected layer: 1024. Because of the high number of neurons in the first fully connected layer, a 80% drop out rate provided the best accuracy (Supplemental Fig. S1d–e). Finally, computing *circa* 8 million features (Supplemental Table S1), our DLBA reached an accuracy of 91.2% (±0.17%) (Fig. 3a). More precisely, the detection precision for “Singles”, Multiples” and “Empty” classes reached 81.7%, 93.2% and 97.6%, respectively. Nevertheless, some images were misclassified, notably for “Singles” and “Death” classes (Supplemental Fig. S1f).

### Classical computer vision and shallow learning-based algorithms

**Figure 2:**
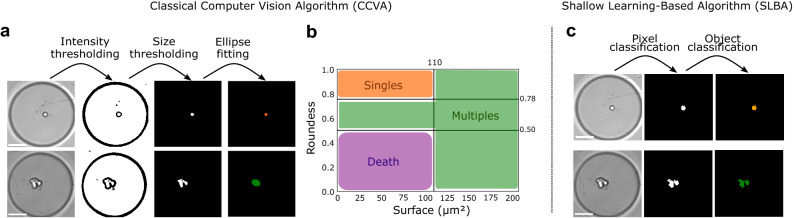
Working principles of the CCVA and SLBA tested in this study. (**a**) For the CCVA, images were processed through an intensity threshold before a size filtering. Remaining objects were fit to an ellipse. First row: processing of a well containing a single cell, second row: processing of a well containing multiple cells. (**b**) According to the area and roundness of the ellipse, images were classified as “Singles”, “Multiples”, “Death” or “Empty”. (**c**) For the SLBA, the first step of pixel classification segments cells from the background. Then, thanks to an object classification tool (Ilastik^27^), images were classified between “Singles”, “Multiples”, “Death” and “Empty” categories. The first row shows the processing of an image classified as “Single”, second row image of well classified as “Multiple”. Colour code corresponds to Fig. 1. Scale bars show 50 μm.

**Figure 3:**
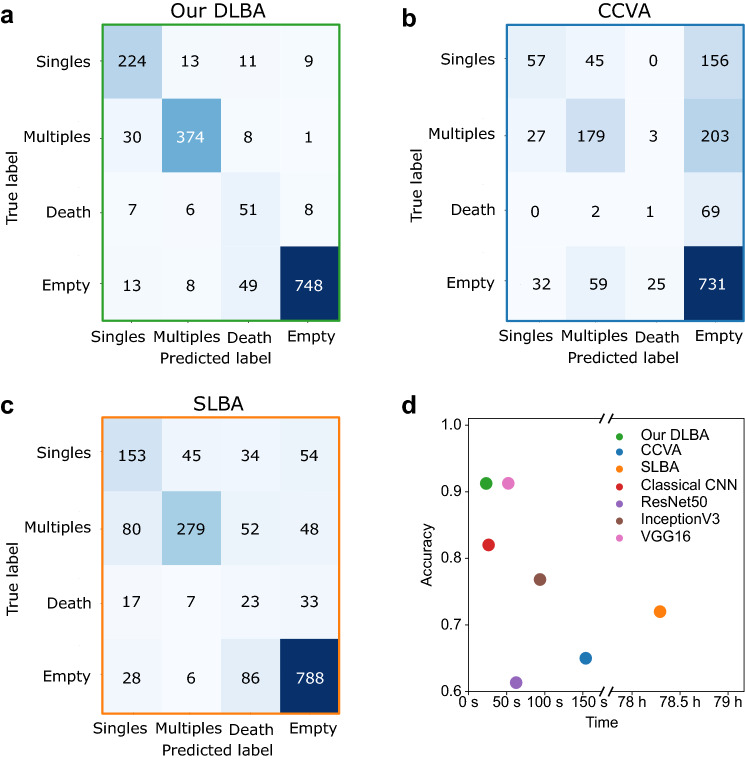
Global accuracy comparison between the different methods tested. (**a**) For our optimized DLBA: Double entry table analysing differences between the ground truth (“True label”, vertical axis) and the prediction (“Predicted label”, horizontal axis). Our optimized DLBA provided an accuracy of $91.2\pm 0.17\%$. Recall and precision were respectively 87% and 81% for “Singles”, 90% and 93% for “Multiple”, 70% and 42% for “Death”, and 91% and 97% for “Empty”. (**b**) The CCVA provided an accuracy of 65%. Recall and precision were respectively 22% and 66% for “Singles”, 42% and 62% for “Multiple”, 1% and 3% for “Death”, and 89% and 63% for “Empty”. (**c**) The SLBA provided an accuracy of 72%. Recall and precision were respectively 51% and 55% for “Singles”, 64% and 81% for “Multiple”, 22% and 10% for “Death”, and 88% and 85% for “Empty”. The color range stands for the amount of images in each category: the darker, the more images are associated. (**d**) Accuracy versus computation times for the 7 methods tested, for 1000 images See Table 1.

To offer a baseline comparison with our optimized DLBA, we also investigated our classification problem with a classical computer vision algorithm (CCVA). We developed a CCVA that pre-processed images in a few steps (Fig. 2a, b: (i) first, objects were segmented according to intensity thresholds, (ii) the largest and smallest segmented objects were filtered depending on their size in pixels, (iii) the remaining objects were fitted to an ellipse, (iv) according to the area and the roundness of the ellipse, the objects were classified as “Singles”, “Multiples”, “Death” or “Empty” if no object was segmented. Five features were analysed by the CCVA: (i) Intensity thresholding was based on the Otsu method^26^, (ii) size filtering has been empirically optimized based on 175 images of validation data set (Supplemental Table S3), (iii) position, as bordering objects were removed, (iv) finally, roundness and (v) area thresholds were optimized among a wide range of values to provide the best accuracy (Supplemental Fig. S4a). When applied to a test data set (Supplemental Fig. S1a, b), the CCVA only provided an accuracy of 65% (Fig. 3b). More precisely, it appeared that a lot of images with single or multiple cells were classified as “Empty” (*circa* 57%), either because they were not segmented properly by intensity thresholding, or their size did not match the size filter. The image-to-image variability is large, more likely due to variations in illumination, cell morphologies, microwell shape and depth of field (Supplemental Fig. S4b) and explains why a CCVA with few features (5) provided a low detection accuracy.

As an additional comparison with our optimized DLBA, a similar analysis was undertaken with a SLBA. Random forest has been shown to be one of the most accurate SLBA^28^. We therefore used a random forest classifier implemented under Ilastik software^27^. Thanks to its pixel classification combined with object classification methods, several studies used this software for image analysis^13^. Based on these studies, we performed a pixel classification step by training the software to segment cells from the background on 40 images from the validation data set (Supplemental Fig. S1a, b and Supplemental Table S3). Then, using an object classification method, segmented objects were classified between “Singles”, “Multiples”, “Death” or “Empty” (Fig. 2c). The SLBA processed respectively 5 and 3 features for pixel classification and object classification steps (Supplemental Table S4). Applied to the test data set (Supplemental Fig. S1a, b), the SLBA performed a better classification than the CCVA: its accuracy reached 72% (Fig. 3b, c). While many images with cells were still classified as “Empty” (*circa* 13%), there were also a lot of misclassifications between “Singles” and “Multiples” classes (*circa* 16%), see Fig. 3c.

### Comparison of our DLBA with CCVA, SLBA and other CNNs

Our optimized DLBA proved to be a more accurate and a much faster (computation time *circa* 20 ms per image) classifier than the CCVA and SLBA previously described (Table 1 and Supplemental Fig. S5).

Our optimized DLBA has also been compared to other cutting edge CNNs (Table 1). For a similar purpose, Anagnostidis et al^15^ developed a simple similar CNN which provided a lower accuracy when applied to the test data set: 82% (±0.26%). We have also trained pre-trained CNNs (VGG16, InceptionV3, classical CNN and ResNet50) on our training data set. Although transfer learning-based algorithms were computing more features and required more computation time, they did not show better accuracy than our optimized DLBA when applied on our test data set (although VGG16 gives close results 91.2% (±0.13%)) but for larger computation times (Fig. 3d).

**Table 1:** Comparison of CCVA, SLBA, our optimized DLBA and other publicly available CNNs.

Algorithm	Machine learning	Deep learning	Transfer learning	Number of features	Accuracy (%)	Computation time per image (s)
CCVA	−	−	−	5	65	$0.15\pm 0.1$
SLBA	$+$	−	−	8	72	$281\pm 36$
Classical CNN^15^	$+$	$+$	−	162,900	$82\pm 0.26$	$0.02\pm 0.03$
ResNet50^20,22^	$+$	$+$	$+$	23,587,712	$61.3\pm 1.53$	$0.06\pm 0.07$
InceptionV3^29^	$+$	$+$	$+$	21,810,980	$78.6\pm 0.06$	$0.09\pm 0.13$
VGG16^21^	$+$	$+$	$+$	17,943,108	$91.2\pm 0.13$	$0.05\pm 0.04$
our DLBA	$+$	$+$	−	8,541,700	$91.2\pm 0.17$	$0.02\pm 0.03$

For the CNNs, we show the mean accuracy ± standard deviation after three model trainings. The mean of computation time per image is given in seconds ± standard deviation.

### Encoding of Temporal information

**Figure 4:**
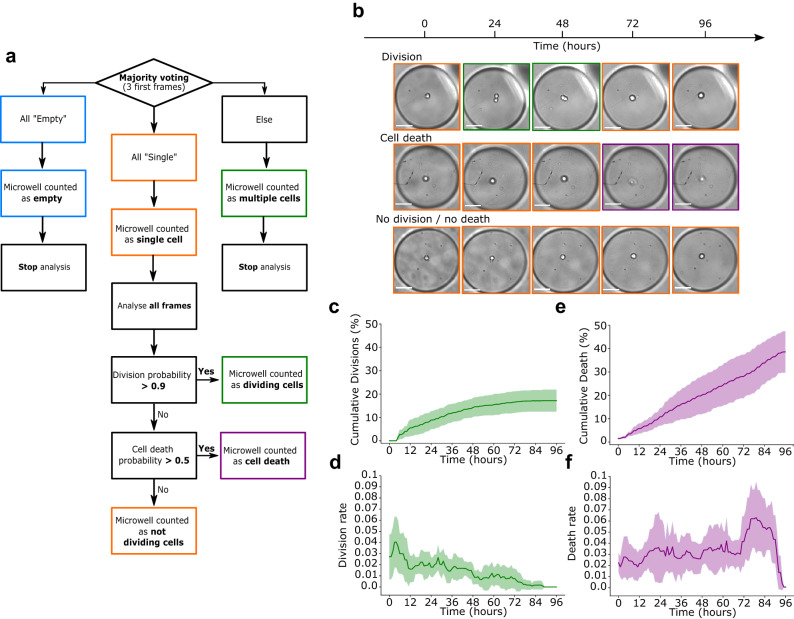
Dynamic analysis with our DLBA. (**a**) Downstream of the CNN, a decision tree considers the temporal information of cell time-lapses in order to detect single cells, cell divisions and cell death. First, we performed a majority voting on the first three frames for each microwell. If all these three frames were classified as “Empty”, the microwell was considered as containing no cell and its time-lapse was not further analysed. Then, if any of these three first frames was classified as “Multiple” or “Death”, the microwell was respectively considered as containing multiple cells or dead cells and was discarded from further analysis too. Finally, if all the three first frames were classified as “Single”, the microwell was considered as containing a single cell and the analysis kept on going. Among each time-lapse with an initial single cell, if two frames were classified as “Multiple” with a probability> 0.9 (of which one>0.99), we considered that a cell division occurred in the microwell. Else, if any frame was classified as “Death” with a >0.5 probability, we considered that a cell death occurred in the microwell. In any other case, we considered that there were still a living yet non-dividing cell in the microwell. (**b**) Examples of Time-Lapse images displaying cell division, death or neither of the two. Scale bars: 50 $\mu$M. Cumulative dynamics of N14-0510 cells divisions (**c**) and cell death (**e**) were computed over time. Relative division rate (**d**) and death rate (**f**) were computed, over sliding 6-h temporal windows. Plots show means and standard deviations, over 4 experiments with three replicates each (2780 time-lapses analyzed in total).

So far, our DLBA analysed only separated 2D images, but to assess the dynamics of single cell fate, temporal information of time-lapses need to be integrated. In addition, we considered that the temporal encoding could rescue some of our DLBA’s misclassification events. Indeed, two cells can adhere tightly to each other in suspension, while having the morphology of a single cell, but detecting events such as cytokinesis can demonstrate that two cells were actually present in the microwell instead of a single fortuitously assumed single cell. We have thus developed a post-analytical decision tree (Fig. 4a). The detection of single cells, cell divisions and cell death with this decision tree was evaluated by computation of recall and precision on fully annotated bright field time-lapses. It appeared that this method very efficiently detected single cells (recall: 93%; precision: 96%), cell divisions (recall: 67%; precision: 94%) and cell death (recall: 82%; precision: 90%) (Time-lapse data set 1, Supplemental Tables S2 and S5). The impact of the frame-rate on the detection of divisions of our DLBA has also been investigated with the time-lapse data set 1. Increasing the interval between two frames induced a lower recall while the precision was not modified (Supplemental Table S6). The probability thresholds and the majority voting can be considered as hyper-parameters that have been empirically tuned. Besides, we have not seen any single cell escaping from its microwell during time-lapses annotation. Hence, we have not considered classification of image as “Empty” during the time-lapse of a microwell if a single cell was detected at the beginning of the acquisition.

In addition, the impact of imaging magnification was also investigated (Time-lapse data set 2, Supplemental Fig. S3 and Table S2). Recall and Precision were computed for two cell lines and showed similar results, although it appeared that the detection of cell death with higher (20$\times$) magnification images was more sensitive while lower magnification (10x) led to a more sensitive detection of cell divisions (See Supplemental Table S2).

### Testing our DLBA on an independant model of CSCs

Although our DLBA was trained on N14-0510 CSCs, we challenged it on the Time-lapse data sets 3 and 4, which were respectively composed of images of N14-1525 cells with 20$\times$ and 10$\times$ magnifications (Supplemental Tables S5). N14-1525 cells are CSCs derived from another independent glioblastoma tumor and cultured as tumorspheres in the same non-adherent conditions as previously described for the N14-0510 cells. Recalls for the N14-1525 cells were overall slightly decreased compared to N14-0510 cells, but the precision of detection remained similar (Supplemental Table S2). N14-1525 cells are larger than N14-0510 cells (Supplemental Fig. S3 and Supplemental Table S7) which may explain the lower recalls. Altogether, we can consider that the global performance remains excellent which suggests that our DLBA can be used on different CSC models.

### Dynamics of cell divisions and cell death using our DLBA

As we compared the accuracy of our DLBA to other CNNs and assessed its robustness to different magnifications and brain CSC models, we next tested its ability to extract relevant biological features of these cells, such as time-resolved division and death rates. We performed 4 experiments with N14-0510 cells (3 replicates each). Brightfield time-lapses were acquired over 96 h, with a 20$\times$ magnification objective, using 80-min imaging intervals. Upon completion, all time-lapses were analysed with our DLBA. The analysis of each experiment took less than 1 h. In total, 2780 single cells were detected and 17% (±5%) underwent cell division within 96 h (Fig. 4b, c). When looking at the dynamic curve of cell divisions, we saw that most divisions occurred during the first 48 h, with a curve flattening after 60 h. Because the low number of late divisions (after 60 h) was overwhelmed by the initial numbers of single cells, computing dynamics did not allow a clear view of the latest divisions. In order to emancipate from the initial number of single cells, we have plotted the instantaneous division rate (Fig. 4b and d). The initial division rate was *circa* 0.03 division/hour, but interestingly, it was decreasing over time. Regarding cell death, 38% (±9%) of single cells died during the 96-h time-lapse (Fig. 4b, e). Compared to cell proliferation rates, the dynamics of cell death rather seemed linear, suggesting that instantaneous death rates should be relatively stable over time. Indeed, the instant death rate was constant *circa* 0.03 death/hour, but it seemed to transiently increase after 72 h (up to 0.07 death/hour) (Fig. 4b, f). Taken together, these results show that for the cell line tested, our DLBA can track automatically single cell fate and follow dynamics of cell phenotypes over time at high throughput rate using brightfield microscopy.

## Discussion

Deep learning-based strategies have been developed to detect or predict cell differentiation. They either rely on morphological transformations of cell populations^22,30,31^, or on the detection of gained or lost expression of biomarkers^32,33^. In this study we propose an original approach, taking advantage of hydrogel microwells to isolate cells in suspension and to recapitulate the initial steps of the tumorsphere formation assay. These growth conditions allow the maintenance of the CSC population from a brain tumor which can be demonstrated by the capacity of tumorspheres-derived CSCs to recapitulate tumor growth when injected in the brain of mouse models^34^. In such microfabricated device, identifying whether or not the cells divide provides us with key features about their stemness potential. In this study, we propose a deep-learning based approach to evaluate the fate (division, death, quiescence) of individual CSCs isolated in microwells, including temporal information encoding for cell dynamics. The complexity of our image data sets can be compared to other similar already published image analysis studies. Chen et al recently used a CNN to predict the formation of spheroids from single breast cancer cells, yet their approach strictly relied on the constitutive expression of the fluorescent mCherry protein in cells^16^. Correlating DNA content and label-free morphological features, Blasi et al developed a SLBA in order to classify cell images at different steps during the cell cycle, but the precision for mitosis detection remained low (*circa* 45%)^35^. More recently, with the same purpose, this team combined bright field images and fluorescent stains and extracted additional features allowing their DLBA to provide improved precisions for mitosis detection (*circa* 70%)^29^. As for cell counting approaches, Anagnostidis et al developed a CNN that was very accurate for counting polyacrylamide beads but which had degraded capacities for cell counting (*circa* 85%)^15^ probably due to complex cell morphological features compare to homogeneous polyacrylamide beads. Concerning the detection of dead cells, Riba et al developed a DLBA in order to distinguish viable cells from dead cells. Interestingly, as we do, they showed that their optimised CNN was more accurate (*circa* 80%) than more complex networks^36^. Here, our DLBA cannot only detect images of microwells containing single cells with high recall and precision (93% and 96% respectively), but it can also detect dividing and dead cells with high reliability all at once, while integrating a time dimension to the analysis. Therefore constituting an innovative and more comprehensive approach to systematic cell phenotyping.

Pre-trained CNNs have already provided excellent results to detect cell death or differentiation for adherent cells on a 2D surface^20–22^. Interestingly, such CNNs did not provide a better accuracy than ours while requiring longer computing times (see Table 1). Transfer learning CNNs have been pre-trained on the publicly available ImageNet database^25^. While it has already been reported that transfer learning did not always improve deep learning performances^37^, other results suggested that CNNs pre-trained on images dedicated to a similar purpose enhance network accuracy^19^. Statistics of natural images are known to produce in the Fourier domain power law spectrum in log-log scales^38^. This means that there are no specific size of objects, but rather objects of all sizes. In our images instead, we have a single object with a given size range, typically a blob which will produce a spectrum with holes (zeros) in the Fourier spectrum. This may explain the relative inefficiency of the pre-trained models applied to our problem. Therefore, in comparison with this most related literature, the imaging and the image analysis have been optimized in order to deliver biologically relevant results with large statistics and high-throughput. Indeed each microwell contains a low number of cells or no cells at the beginning of the experiment. Consequently, the complexity of the informational task is reduced and this accounts for the much simpler and smaller neural network that we obtained as the best solution. Therefore, the use of the microwell systems not only increases the experimental throughput but also reduces the computational complexity of the neural network. From a methodological point of view, our CNN architecture has demonstrated to provide better results than standard architectures with a low number of hyperparameters to be tuned. Our approach enables easy time dependent processing and is more compatible with on-the-fly analysis. This is obtained thanks to the joint optimization of hardware (microwell approach) and software (small CNN) which results in a globally more efficient solution.

Although our DLBA provided good predictions for a given cell line while trained on a different one. However, a thorough examination to additional cell lines is still necessary to generalize the improved capacities of our method, yet when suspended in microwells, cells typically take on a spherical form and don’t display much variability in shape compared to adherent cells. Indeed, misclassification seems to results from differences in the mean size of each cell line (Supplemental Fig. S3 and Supplemental Table S7). An efficient way to improve performances of the CNN could be adding to our image data sets more images from different cell lines with various sizes or textures, i.e. cell models derived from other patients or tissues. Although this annotating step require some more work, it would be still less demanding than programming a new CCVA optimised to each new cell line. Another very interesting perspective would be to implement temporal convolution to our CNN in order to better take into account the temporal dimension of time-lapses. Despite implying the re-annotation of the training datasets used so far, the analysis of time series by CNNs should allow to significantly improve the prediction of cell fate^39^, for example by predicting cell divisions before it happens based on cell morphological characteristics.

We have also investigated the dynamics of cell division and death of CSCs originated from glioblastoma. These glioblastoma CSCs are at the centre of controversies because of the lack of reliable molecular markers to specifically identify them^4^. Morever, recent single cell transcriptomic data suggest that glioblastoma tumor-forming cells are rather defined by a continuum of cellular states, including different CSC phenotypes and cells engaged towards more differentiated cancer cells populations^40–42^. Similarly, although Patel et al suggested that CSCs express cell cycle related genes at low level, suggesting that these cells likely have low division rates, other recent single RNA-seq studies conversely suggest that glioblastoma stem or progenitor-like cells are enriched in cycling cells^43^. These conflicting results further support the need to develop approaches, such as the one proposed in this study, to further describe or experimentally assess hypotheses regarding the behavior of CSCs. Accordingly, this heterogeneity between slow and fast dividing cells could be recapitulated in Fig. 4c and might arise from cells unequally positioned on the continuum of differentiation phenotypes. Besides, CSCs have been shown to escape anoïkis^44^, and more specifically, N14-0510 cells have been reported to display an increased expression of anti-apoptotic factors under non-adherent culture conditions^45^. Our microwells prevent cell-substrate adhesion as well as cell-cell adhesion, therefore suggesting that cells remaining alive at the end of the time-lapse might recapitulate several CSC properties, including survival of isolated cells and tumorigenic capacities highlighted by the formation of tumorspheres. To further prove the correlation between cell state, early division and fate, it would be interesting to monitor stemness or differentiation molecular markers i.e. OLIG2, SOX2 or GFAP in our microwells and correlate their expression with late events such as tumorsphere formation. This would provide unique information on the dynamics of CSCs linked to biological functions. Finally, coupling our DLBA to a micro-fabricated device dedicated to drug screening would provide a relevant image analysis pipeline in order to assess, on-the-fly and at high throughput rate, drug effects on cell divisions, cell death and CSCs fate modifications for patient-derived dissociated tumor samples.

## Methods

### Microfabricated device

Manufacturing process has already been detailed in Goodarzi et al^23,24^. Briefly, 200 $\mu$m diameter microwells were molded with 2% agarose solution over PDMS counter-moulds. Agarose was then immobilized on (3-Aminopropyl)triethoxysilane coated glass cover-slips. Eventually, GSCs were seeded in the agarose microwells.

### Cell lines and culture

N14-0510 and N14-1525 cell lines were kindly provided by A.I. and M.G. labs and were derived from diagnosed WHO grade IV glioblastoma before established as cellular models maintain in non-adherent conditions. They were maintained under normoxia at 37$^{\circ }$C in incubator, in Dulbecco’s modified Eagle’s medium/nutrient mixture F12 (Life, 31330-095) complemented with N2 (Life, 17502-048) at 1X, B27 (Life, 17504-001) at 1X, 100 U/ml penicillin-streptomycin (Life, 15140-122) and FGF2 (Miltenyi Biotec, 130-104-922), EGF (Miltenyi Biotec, 130-093-825) (20ng/mL both) and heparin 0.00002% (Sigma, H3149). Unless otherwise specified, cells were cultured in Ultra low attachment T75 flasks (ThermoFisher, Ultra Low Adherent, 10491623). Cells were passaged weekly with Accumax (Sigma, A7089) at a density of 600 000 cells in 20 ml of complete media. The medium was renewed twice a week and mycoplasma tests were regularly performed. Hoechst staining (Sigma, H6024) was used to control cell number (10 and 100 ng/mL) and TO-PRO-1 iodide (ThermoFicher, T3602) was used to control cell viability (1:50). Cell size measurements were performed after enzymatic dissociation of tumorsphere as previously detailed, the cell suspensions were quantified using the automatic LUNA FL cell counter (Logos biosystems) following the manufacturer’s instructions. Briefly, cells were stained with acridine orange and propidium iodide stain solution and were immediatly imaged with both brightfield and dual fluorescence optics to discriminate dead from living cells, and to estimate the size of living cells.

### Image setup

Samples were images with a Leica DMIRB microscope. Microscope was located in a impervious box with 37$^{\circ }$C controlled temperature (LIS Cube) and 5% CO2 air (LIS Brick gaz mixer). The camera (Andor Neo 5.5 SCMOS), shutter (Vincent associated D1) and stage (Prior proscan II) were controlled with micromanager 1.4.22^46^. The light source was provided by a LED (Thorlabs MWWHL4). 20$\times$ and 10$\times$ numerical aperture objectives were both from Leica.

### Hardwares and softwares

Computing was performed with Windows 10, 64 bit operating system, Intel(R) Core(TM) i7-7700 3.60GHz processor. GPU used was NVIDIA quadro p600. All scripts were written in Python 3.7^47^. Libraries used were Mahotas 1.4.11^48^, OpenCV 4.2.0^49^, Seaborn^50^, numpy^51^, SciPy^52^, pandas^53^, matplotlib^54^ and Tensorflow 2.3.0^55^. Version of Ilastik used was 1.3.2post1^27^. Pre-trained CNN were found at https://www.tensorflow.org/api_docs/python/tf/keras/applications.

### Data sets and code availability

Annotated data set was composed of 17,378 bright field acquired images. Images have been manually annotated. All cells from this data set were N14-0510 cells imaged with a 20$\times$ magnification. Amount of images per class was: 2871 “Singles” images, 4615 “Multiples” images, 803 “Death” images and 9089 “Empty” images (Fig. 1b and Supplemental Fig. S1a). The number and viability of cells seen in brightfield has been controlled by fluorescent microscopy (Supplemental Fig. S2). 10% of these images were randomly selected in order to generate a validation data set, and another 10% was also randomly selected for the test data set. Remaining images constitute the training data set, on which we performed data augmentation in order to balance number of images between the four classes (Supplemental Fig. S1b). Parameters of SLBA and CCVA were optimized respectively with 40 and 175 images manually selected from validation data set (Supplemental Table S3). Performance of DLBA, SLBA and CCVA were all compared on test data set. Computation times were compared on 1, 10, 100 and 1000 images randomly selected from test data set. Time-lapses were performed with a time interval of 40 min (Supplemental Table S6), except for the dynamics of cell division and death (Fig. 4) where a 30 min interval was used. Time-lapse data set 1 was composed of 1091 annotated time-lapses of N14-0510 cells imaged with 20$\times$ magnification. There were 356 empty microwells, 434 microwells with 2 cells or more, 301 microwells with single cells which 81 divided and 117 died. Time-lapse data set 2 was composed of 1179 annotated time-lapses of N14-0510 cells imaged with 10x magnification. There were 363 empty microwells, 482 microwells with 2 cells or more, 334 microwells with single cells which 71 divided and 85 died. Time-lapse data set 3 was composed of 717 annotated time-lapses of N14-1525 cells imaged with 20$\times$ magnification. There were 231 empty microwells, 310 microwells with 2 cells or more, 176 microwells with single cells which 26 divided and 34 died. Time-lapse data set 4 was composed of 596 annotated time-lapses of N14-1525 cells imaged with 10x magnification. There were 222 empty microwells, 214 microwells with 2 cells or more, 160 microwells with single cells which 31 divided and 55 died. Image databases and codes can be found at https://github.com/chalbiophysics/XXX.

### Statistics

Classical efficiency scores were performed to evaluate and compare algorithms. Those scores involve true positives (positive images correctly classified, or TP), true negatives (negative images correctly classified, or TN), false positives (negative images misclassified, or FP) and false negatives (positive images misclassified, or FN). Accuracy was computed when CCVA, SLBA and the various CNNs:

1 $$\begin{aligned} Accuracy = \frac{TP + TN}{TP+TN+FP+FN}. \end{aligned}$$

When assessing time-lapse classification and comparison between cell lines and magnifications by DLBA, recall and precision were computed:

2 $$\begin{aligned} Recall= & {} \frac{TP}{TP+FN}, \end{aligned}$$

3 $$\begin{aligned} Precision= & {} \frac{TP}{TP+FP}. \end{aligned}$$

Numpy library^51^ was used to compute means and standard deviations. Percentages of cell divisions and cell death through time were computed as follows:

4 $$\begin{aligned} \%events_{time} = 100 * \frac{N\,cell\,events_{time}}{N\,single\,cells}, \end{aligned}$$

$\%events_{time}$ is the percentage of cell division or death at given time point; $N\,cell\,events_{time}$ are number of cell divisions or death at given time; $N\,single\,cells$ is the initial number of single cells at beginning of time-lapse. Division rate and death rate through time were computed upon a 6-h temporal window:

5 $$\begin{aligned} Event\,rate_{time} = \frac{N\,cell\,events_{time} - N\,cell\,events_{time - 6 h}}{N\,living\,single\,cells_{time}}, \end{aligned}$$

$Event\,rate_{time}$ is the division or death rate at given time, $N\,cell\,events_{time}$ are the number of cell divisions or death at given time, $N\,living\,single\,cells_{time}$ is the remaining number of single cells that are still alive and have not divided yet at given time.

## Supplementary Information

Supplementary Information.
