# Supplementary material for: Machine learning-based detection of label-free cancer stem-like cell fate
Source: Sci Rep. 2022 Nov 9;12:19066. doi: 10.1038/s41598-022-21822-z (PMC9646748; doi:10.1038/s41598-022-21822-z)
Supplement: Supplementary file 1 — Supplementary Information. [file 41598_2022_21822_MOESM1_ESM.pdf]

Supplementary information

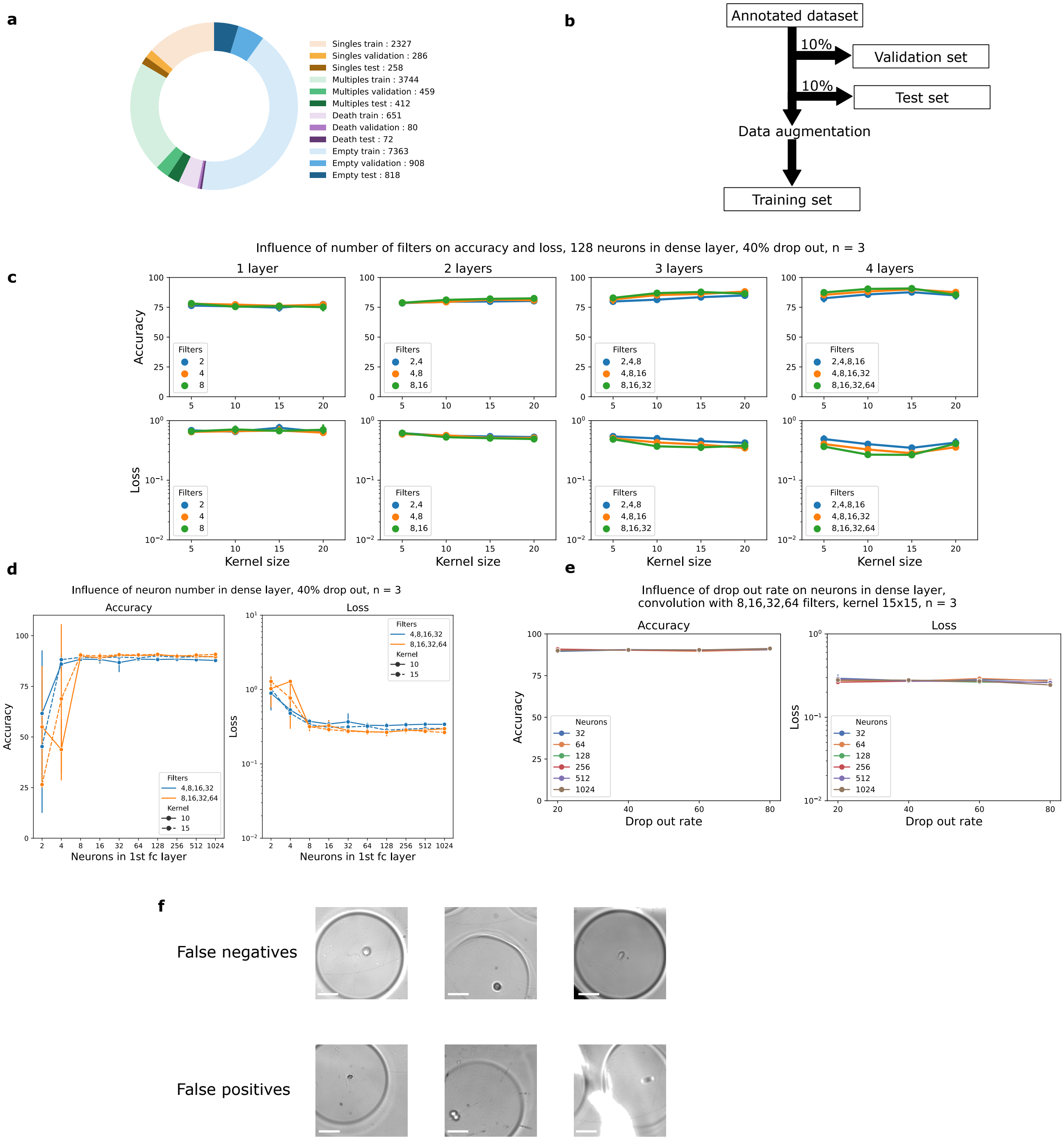

Figure S1. DLBA databases and optimization. (a) 17378 images were manually annotated between “Singles”, “Multiples”, “Death” and “Empty” classes. (b) 10% of the image data set constituted the validation set and another 10% was used as a test set. We performed data augmentation (clockwise 90° rotation, clockwise -90° rotation, 180° rotation, horizontal flip and vertical flip) in order to balance the classes. We optimized the kernel size, number of layers and number of filters per layer (c), number of neurons in first fully connected layer (d) and dropout rate (e). Plots show means and standard deviations of three model trainings. (f) Examples of images from “Singles” class misclassified. Scale bars show 50 µm.

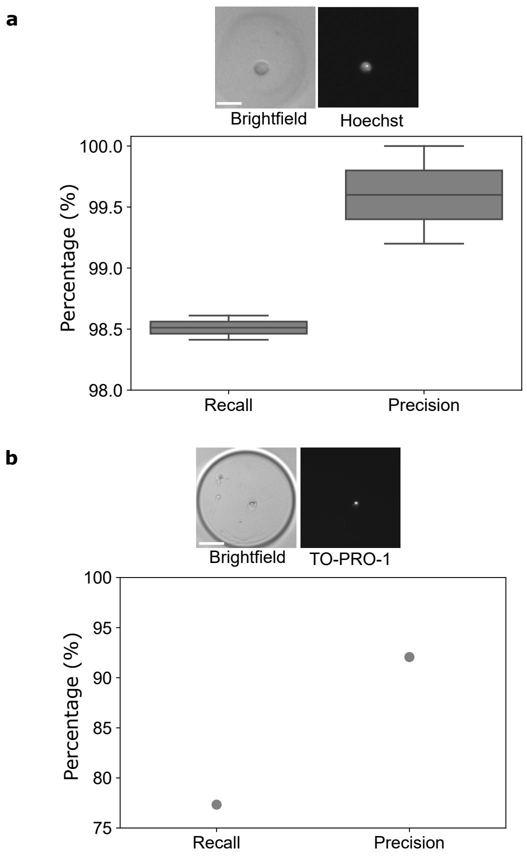

Figure S2. Fluorescence labeling validation of the database of brightfield images. (a) Single cell counting was controlled with Hoechst staining and fluorescence imaging (100 ng/mL). For box plot n=777 analysed images (b) Cell viability was assessed by TO-PRO-1 iodide staining, fluorescence imaging and analysis of 1069 images. Scale bars show 50 µm.

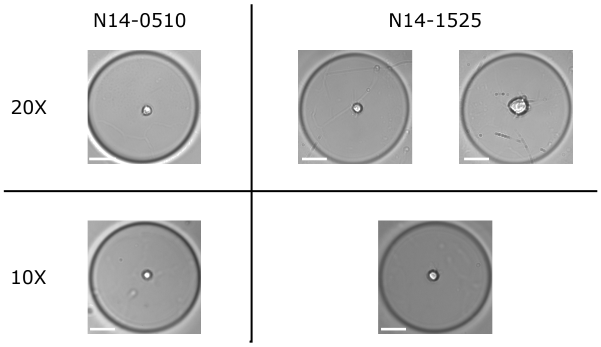

Figure S3. Examples of N14-0510 and N14-1525 cells imaged with 20x and 10x magnification, mean diameters are respectively $14.8 \pm 0.5 \mu m^3$ and $17 \pm 0.6 \mu m^3$ (see Supplemental Table S7). Scale bars show 50 µm.

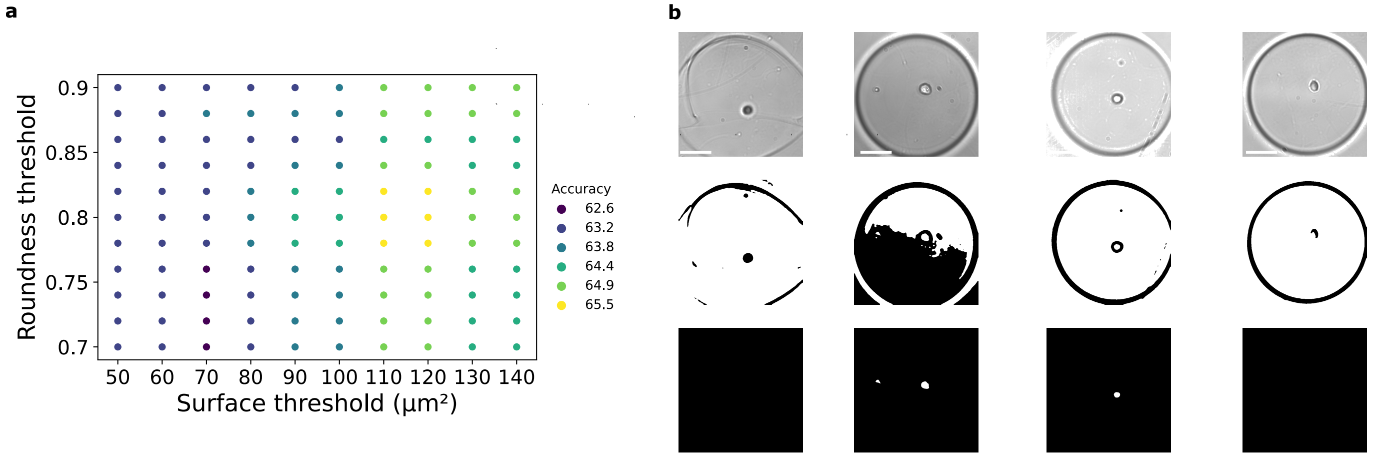

Figure S4. CCVA optimization and misclassifications. (a) Roundness and area thresholds were selected in order to optimize accuracy of the classification problem. (b) First row illustrates variability of images taken with brightfield microscopy. Second row: Intensity thresholding of corresponding images and third row: results of ellipse fitting. Scale bars show 50 µm.

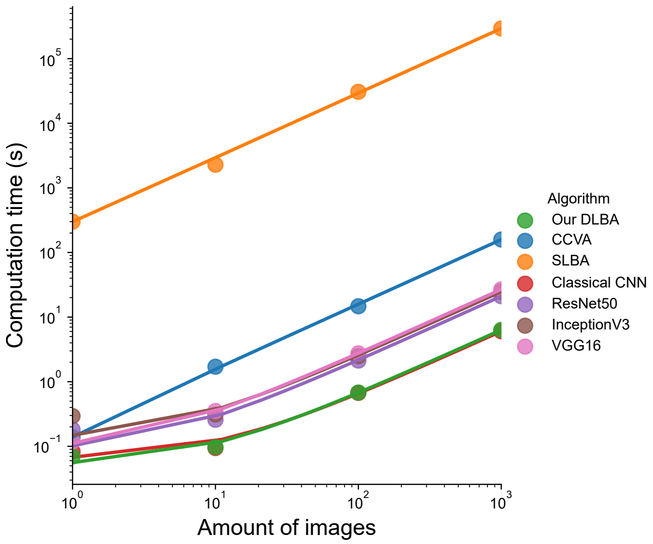

Figure S5. Comparison of the different algorithms computation times. We compared the computation time needed to analyse 1, 10, 100 and 1000 images with CCVA, SLBA, DLBA, an already published classical CNN[1], ResNet50, InceptionV3[2] and VGG16[3].

Layer (type	Output Shape	Number of features
conv2d (Conv2D)	(None, 285, 285, 8)	1808
max_pooling2d (MaxPooling2D)	(None, 142, 142, 8)	0
conv2d_1 (Conv2D)	(None, 128, 128, 16)	28816
max_pooling2d_1 (MaxPooling2D)	(None, 64, 64, 16)	0
conv2d_2 (Conv2D)	(None, 50, 50, 32)	115232
max_pooling2d_2 (MaxPooling2D)	(None, 25, 25, 32)	0
conv2d_2 (Conv2D)	(None, 11, 11, 64)	460864
fatten (Flatten)	(None, 7744)	0
dense (Dense)	(None, 1024)	7930880
activation (Activation)	(None, 1024)	0
dropout (Dropout)	(None, 1024)	0
dense_1 (Dense)	(None, 3)	3075
activation_1 (Activation)	(None, 3)	0

Table S1. Features computed by our optimized DLBA.

		N14-0510		N14-1525	
	**Magnification**	**Recall**	**Precision**	**Recall**	**Precision**
**Single cell**	20x	93% (281/301)	96% (281/292)	80% (142/176)	92% (142/153)
	10x	93% (312/334)	99% (312/314)	86% (138/160)	97% (138/142)
**Division**	20x	67% (55/81)	94% (55/58)	79% (23/28)	88% (23/26)
	10x	80% (57/71)	93% (57/61)	93% (29/31)	87% (29/33)
**Cell death**	20x	82% (96/117)	90% (96/106)	54% (27/50)	79% (27/34)
	10x	70% (60/85)	89% (60/67)	65% (36/55)	92% (36/39)

Table S2. Recall and precision for the detection of time-lapses with one single cell, of cell divisions and of cell death. The table shows results in percentage and absolute numbers for N14-0510 cells at 20x and 10x magnifications, and for N14-1525 cells at 20x and 10x magnifications (respectively 1091, 1179, 717 and 596 time-lapses analysed).

Algorithm	Data sets used for parameters optimization
DLBA	Training set & Validation set
SLBA	40 images from Validation set
CCVA	175 images from Validation set

Table S3. Our DLBA was trained on Training set and weights were optimized based on Validation set. SLBA was trained on 40 imaged manually selected from "Singles", "Multiples" and "Death" classes from Validation set. CCVA was trained on 10% of images from each classes of Validation set. All algorithms were tested with the Test set.

Pixel classification
Gaussian smoothing (σ 10)
Gaussian gradient magnitude (σ 5)
Difference of gaussians (σ 0.7)
Structure tensor eigenvalues (σ 1.6 and 10)
Hessian gaussian eigenvalues (σ 3 and 10)
**Object classification**
Sizing in pixels
Convexity
Average Branch length

Table S4. Features computed by ilastik for pixel classification and object classification steps.

Time lapse data set #	Cell line	Magnification	Amount of time-lapses annotated	Micro-wells with a single cell	Micro-wells with a cell division	Micro-wells with a cell death
1	N14-0510	20x	1091	301	81	117
2	N14-0510	10x	1179	334	71	85
3	N14-1525	20x	717	176	26	34
4	N14-1525	10x	596	160	31	55

Table S5. Four time-lapse data sets were manually annotated between empty micro-wells, micro-wells with multiple cells at beginning of experiment, micro-wells with a single cell at beginning of experiment, micro-wells with a cell division and micro-wells with a cell death event. Both N14-0510 and N14-1525 cell lines were imaged with 10x and 20x magnification.

	Time interval (min)		
	**40**	**80**	**120**
Recall	84%	56%	41%
Precision	94%	97%	100%

Table S6. Impact of the time interval between two frames on the performances of our DLBA. All results were computed with the time-lapse data set 1 from which 1 frame out of 2 was removed for 80 min time intervals and 2 frames out of 3 were removed for 120 min intervals.

	Mean diameter (μm)	
N14-0510	$14,86 \pm 0,5$	(n= 2830)
N14-1525	$17,06 \pm 0,53$	(n= 2246)

Table S7. Mean diameter for N14-0510 and N14-1525 cell lines. Measurements were performed with a commercial automated cell counter (See Methods)

References

1. Anagnostidis, V. *et al.* Deep learning guided image-based droplet sorting for on-demand selection and analysis of single cells and 3D cell cultures. *Lab on a Chip* **20**, 889–900, DOI: 10.1039/D0LC00055H (2020). Publisher: The Royal Society of Chemistry.

2. Eulenberg, P. *et al.* Reconstructing cell cycle and disease progression using deep learning. *Nat. Commun.* **8**, 463, DOI: 10.1038/s41467-017-00623-3 (2017). Number: 1 Publisher: Nature Publishing Group.

3. Verduijn, J., Van der Meeren, L., Krysko, D. V. & Skirtach, A. G. Deep learning with digital holographic microscopy discriminates apoptosis and necroptosis. *Cell Death Discov.* **7**, 1–10, DOI: 10.1038/s41420-021-00616-8 (2021). Number: 1 Publisher: Nature Publishing Group.
